# Genotoxicity of Tri- and Hexavalent Chromium Compounds *In Vivo* and Their Modes of Action on DNA Damage *In Vitro*

**DOI:** 10.1371/journal.pone.0103194

**Published:** 2014-08-11

**Authors:** Zhijia Fang, Min Zhao, Hong Zhen, Lifeng Chen, Ping Shi, Zhiwei Huang

**Affiliations:** 1 College of Chemistry, Chemical Engineering and Biotechnology, Donghua University, Shanghai, China; 2 State Key Laboratory of Bioreactor Engineering, East China University of Science and Technology, Shanghai, China; University of Kentucky, United States of America

## Abstract

Chromium occurs mostly in tri- and hexavalent states in the environment. Hexavalent chromium [Cr(VI)] compounds are extensively used in diverse industries, and trivalent chromium [Cr(III)] salts are used as micronutrients and dietary supplements. In the present work, we report that they both induce genetic mutations in yeast cells. They both also cause DNA damage in both yeast and Jurkat cells and the effect of Cr(III) is greater than that of Cr(VI). We further show that Cr(III) and Cr(VI) cause DNA damage through different mechanisms. Cr(VI) intercalates DNA and Cr(III) interferes base pair stacking. Based on our results, we conclude that Cr(III) can directly cause genotoxicity *in vivo*.

## Introduction

Chromium (Cr) is a heavy metal and a well-known environmental contaminant mainly exists in hexavalent [Cr(VI)] and trivalent [Cr(III)] states [1]. High levels of exposure to Cr(VI) occurs in chromate manufacturing, chrome plating, ferrochrome production and stainless steel welding. Occupational exposure to Cr(VI) compounds is a well-documented cause of respiratory cancers [2], [3], and Cr(VI) is a proven toxin, mutagen and carcinogen [4]–[9]. Many types of structural genetic lesions produced by Cr(VI) have been observed *in vivo* and *in vitro*, including interstrand cross-links, DNA protein cross-links, strand breaks and Cr-DNA adducts [10]–[14]. Association with the phosphodiester backbone of DNA (chromium-DNA adduct) is one of the most abundant genetic lesions induced by chromium in mammalian cells and is thought to be a primary cause of Cr(VI) mutagenicity [7]. However, the molecular mechanism of chromium carcinogenicity is still unclear.

In contrast, Cr(III) was initially considered a relatively nontoxic agent that plays an important role in regulating blood glucose levels and is regarded as a dietary supplement [15]–[18]. Studies in cell-free systems demonstrated that Cr(III) does bind to DNA, leading to a decrease in the fidelity and an increase in the processivity of DNA polymerases, which may ultimately lead to increased mutations [19]. However, a mutagenic effect of Cr(III) *in vivo* remains to be investigated. It is also unclear whether Cr(III) and Cr(VI) act on DNA through the same or different mechanisms.

In this study, we compared the effects of Cr(VI) (i.e., CrO_3_)and Cr(III) (i.e., CrCl_3_) on DNA damage both *in vivo* and *in vitro*. We found that they both increase mutational rates and cause DNA degradation. However, we found that CrCl_3_ is surprisingly more genotoxic than CrO_3_ in both yeast and animal cells. We also found that these two compounds interact with DNA differently. CrO_3_ binds to DNA in an intercalative manner and irreversibly destroys the configuration of DNA, In contrast, CrCl_3_ interferes with the stacking mode of the base pairs. Taken together, our results suggest that both trivalent and hexavalent chromium compounds are genotoxic and that they cause DNA damage through different modes of action.

## Materials and Methods

### Chemical Reagents

Chromium trioxide (CrO_3_) and chromium chloride (CrCl_3_) were purchased from Sigma. Stock solutions were freshly prepared before each experiment in sterile water. In all experiments, an equal volume of water was used as the vehicle control.

### Mutational Rate Analysis in Yeast

The *S. cerevisiae* strain used in this work was SJR576 (*MATa ade2-1oc can1-100oc leu2-K lys2-1oc ura3ΔNco*). *ade2-1oc*, *can1-100oc* and *lys2-1oc* are ochre alleles of the respective genes suppressible by *SUP4-o*, an ochre suppressor allele of the yeast tyrosine tRNA gene. This *SUP4-o* allele is carried on pRS179, a centromeric vector that mimics chromosome behavior in yeast cells [20]. This plasmid was transformed into SJR576 to generate strain SJR576-p.

The yeast growth media was a synthetic complete medium lacking uracil (SC-Ura). The media for selecting *sup4-o* mutants was a minimal medium consisting of yeast nitrogen base without amino acids (1.5 g/L), ammonium sulfate (5 g/L), glucose (20 g/L), leucine (0.262 g/L), lysine (0.182 g/L), adenine (6.65 mg/L) and canavanine (60 mg/L). YEPD medium containing yeast extract (10 g/L), bacto peptone (20 g/L), dextrose (20 g/L) and adenine (250 mg/L) was used to determine the total number of viable cells being assayed in each experiment.

SJR576-p yeast cells were inoculated in liquid SC-Ura and grown at 30°C with shaking for an overnight. The starter culture was used to inoculate 3 mL fresh liquid SC-Ura with an initial cell density of 2×10^6^ cells/mL. The cells were then incubated in the presence or absence of 300 µM CrO_3_ or 150 µM CrCl_3_ for 24 hours, diluted, and plated on a minimal medium to select for *sup4-o* mutants. The plates were incubated at 30°C for three days and placed at 4°C for about 20 days. Red colonies that emerged on canavanine-containing medium were scored as *sup4-o* mutants. The mutation frequency for each independent culture was determined by calculating the percentage of red and canavanine-resistant colonies of all colonies grown on YPD medium.

### Cell Culture

The human T cell leukemia Jurkat cells were obtained from the Type Culture Collection of the Chinese Academy of Sciences (Shanghai, China). They were cultured in RPMI 1640 supplemented with 10% FBS (Sigma), glutamine (2 mM), penicillin (100 U/ml), and streptomycin (100 µg/mL) at 37°C in humidified air supplemented with 5% CO_2_.

### Single-Cell Gel Electrophoresis (SCGE) Assay

The *S. cerevisiae* strain BY4741 (*MAT* ***a*** *his3Δ0 leu2Δ0 met15Δ0 ura3Δ0*) was treated with 300 µM CrO_3_ or 150 µM CrCl_3_ for 24 hours. The Jurkat cell line was treated with 150 µM CrO_3_ or 150 µM CrCl_3_ for 24 hours. Slides were prepared in duplicate per sample. Fully frosted microscopic slides were covered with 0.8% normal melting agarose (NMA). After the application of a coverslip, the slides were allowed to gel at 4°C for 10 minutes. In the meanwhile, cell suspensions (2×10^6^ cells/mL) were added to 0.65% of low melting agarose (LMA). After carefully removing the coverslips, a second layer of the sample mixture was added onto the pre-coated slides and allowed to solidify at 4°C for 10 minutes. The cover slips were removed, and a third layer of LMA was added onto the slides and allowed to gel at 4°C for 10 minutes.

A slide was immersed in freshly prepared cold lysis solution (2.5 M NaCl, 100 mM Na_2_EDTA, 10 mM Tris-HCl, pH 10, 1% sodium N-lauroyl sarcosinate, 1% Triton X-100 and 10% DMSO [added just before use]) and refrigerated for 1 hour. The slide was then placed in alkaline buffer (300 mM NaOH and l mM EDTA, pH 13) for 30 minutes to allow unwinding of DNA. Electrophoresis was conducted for 30 minutes at 18 V. The slide was then drained, placed on a tray and washed slowly three times with 1xPBS buffer every 5 minutes. DNA was precipitated and the slide was dehydrated in absolute methanol for 10 minutes and before being left at room temperature to dry. Finally, the slide was stained with ethidium bromide and visualized under fluorescent microscope (Olympus BX51, Tokyo, Japan).

### Degradation of Plasmid DNA Induced by Chromium

Supercoiled YEplac195 plasmid DNA was purified from DH5α cells using an EndoFree Plasmid Kit (QIAGEN, USA). Purified plasmid was treated with different concentrations of CrO_3_ or CrCl_3_ at 37°C for 2 hours. All samples were resolved with agarose (1%) electrophoresis to monitor DNA cleavage/degradation induced by the chromium compounds. Agarose gel images were analyzed with the Tanon-3500 gel imaging system (Tanon Science & Technology Co., Ltd. Shanghai, China).

Supercoiled plasmid DNA was also digested with the linearized by endonuclease HindIII. Linearized DNA was incubated in the absence or presence of various concentrations of CrO_3_ or CrCl_3_ as described above. The cleavage products were extracted with a DNA extraction kit (TIANGEN Midi Purification Kit, China) and subjected to ligation reactions using T4 DNA ligase.

To evaluate the effects of buffer and temperature on Cr-induced DNA degradation, CrO_3_ and CrCl_3_ were dissolved in Tris-HCl buffer (0.1M) or PBS buffer (0.5M) of different pH. Purified plasmid DNAwas incubated with CrO_3_ and CrCl_3_ at 37°C for 2 hours at indicated pH or indicated temperature (i.e., 0, 20, 30, and 37°C). To evaluate the effects of DTT on Cr-induced DNA degradation, purified plasmid DNA was treated with DTT (10 mM) or chromium solution (80 µM) containing different concentrations DTT (i.e., 0.1, 0.5, 1.0, 5.0 and 10 mM) at 37°C for 2 hours.

### Circular Dichroism Measurement

Circular dichroism (CD) spectroscopy was performed using a Chirascan CD spectrometer equipped with a temperature-controlled water bath (Applied Photophysics, Leatherhead, UK). A sample was loaded into a 10 mm quartz cuvette. A spectrum was recorded from 200 to 320 nm with a 1 nm bandwidth at 22°C. Each spectrum was averaged from five successive accumulations at a scan rate of 50 nm/minute.

### Fluorescence Competition Binding Assay

A DNA sample (6 µg/mL) was first incubated with or without CrO_3_ or CrCl_3_ (150 µM) and then with an equal volume of ethidium bromide (12 µg/mL). The fluorescence intensity was measured at an excitation wavelength at 520 nm and an emission wavelength at 610 nm using a Genios multifunction-reader (Tecan GENios Pro, Tecan Group Ltd. Maennedorf, Switzerland).

### Melting Temperature-Based SYBR Green I Assay

Total RNA from SH-SY5Y cells was extracted using RNAiso Plus (Takara). The PCR forward and reverse primers for amplifying GAPDH were 5′-AGAAGGCTGGGGCTCATTTG-3′ and 5′ -AGGGGCCATCCACAGTCTTC-3′, respectively. Real-time RT-PCR was performed using the primeScript RT reagent kit with gDNA Erase and SYBR Premix Ex Taq kit (Takara) in a CFX9 Real-Time PCR Detection System (BioRad, Hercules, CA). The products were treated with 300 µM CrO_3_ or 150 µM CrCl_3_. Melting of DNA was performed from 60 to 95°C at 0.1°C/s with a smooth curve setting. The melting peaks were visualized by plotting the first derivative against the melting temperature. A melting temperature (*Tm*) was defined as the peak of the curve.

## Results and Discussion

### Both Cr(VI)and Cr(III) Induce Mutations in Yeast

**Figure 1:**
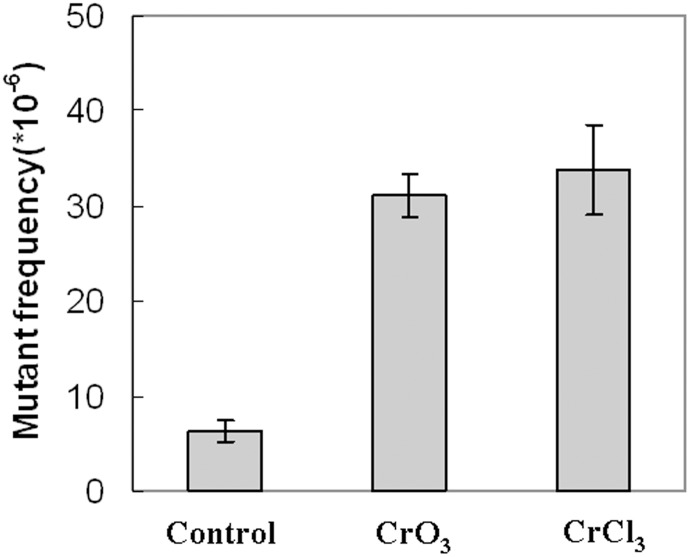
Both Cr(VI) and Cr(III) induce mutations in yeast. Cells of the yeast strain SJR576 carrying the *SUP4-o* plasmid were treated with or without CrO_3_ or CrCl_3_ for 24 hours and plated on indicator plates to select for *sup4-o* mutants. Mutational rates were calculated and plotted. The results are the means of three independent experiments. The error bars represent the means ± SD.

To evaluate the potential effects of Cr(III) and Cr(VI) in causing mutations, we assayed the well-defined *SUP4-o* allele. This allele encode a mutant tRNA that suppresses ochre stop codons by inserting a tyrosine. Two ochre alleles, *ade2-1oc* and *can1-100oc*, were used to monitor the loss of *SUP4-o* function. The *ade2-1oc* mutation causes adenine auxotrophy as manifested as red colony color. The *can1-100oc* mutation causes resistance to cananvanine. The presence of a functional *SUP4-o* allele renders cells containing the *ade2-1oc* and *can1-100oc* form white colonies that are sensitive to canavanine. Mutations that inactivate *SUP4-o* can be identified by the simultaneous loss of suppression of both *ade2-1* and *can1-100* alleles, resulting in red and canavanine resistant colonies. Using this system, we tested whether Cr(VI) and Cr(III) might increase mutational frequency in yeast. In untreated cells, the frequency of loss of *SUP4-o* function was about 6.32×10^−6^. In CrO_3_ (at 300 µM) and CrCl_3_ (at 150 µM) treated cells, the frequency was increased to 31.6×10^−6^ and 33.86×10^−6^, respectively (Fig. 1). Therefore, Cr(VI) and Cr(III) significantly induce the loss of *SUP4-o* function in yeast cells (P<0.001) (Fig. 1).

### Both Cr(VI) and Cr(III) Induce DNA Damage in Yeast and Jurkat Cells

**Figure 2:**
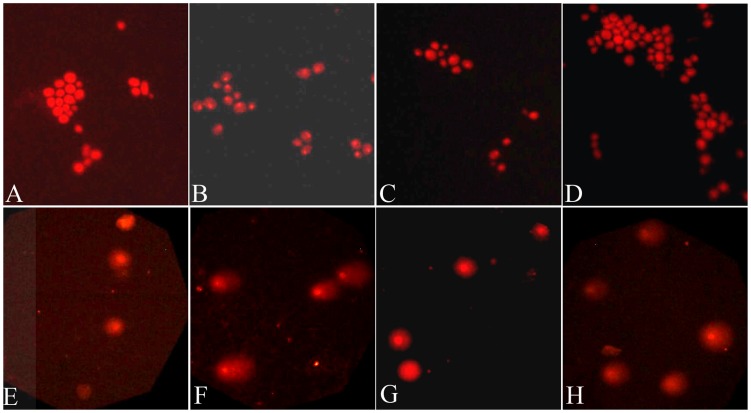
Chromium compounds induce DNA damage in yeast (A–D) and Jurkat cells (E–H). (A, E) Cells were not treated with chromium or a DNA damage treatment. (B) Cells were treated with 20 µM H_2_O_2_ for 30 minutes. (C, G) Cells were treated with 300 µM CrO_3_ for 24 hours. (D, H) Cells were treated with 150 µM CrCl_3_ for 24 hours. (F) Cells were exposed to UV for 1 hour. The slides were viewed using a fluorescent microscope at magnification 200× (A–D) and 100× (E–H).

To explore the possibility that the loss of *SUP4-o* function induced by Cr was at least partly due to DNA damage, we employed a SCGE (or Comet) assay in both yeast and Jurkat cells. In this assay, increased DNA damage is manifested as enlarged comet tails as seen in the positive controls (i.e., hydrogen peroxide in yeast cells and UV irradiation in Jurkat cells) (Fig. 2). We observed increased DNA damage in samples treated with either CrO_3_ or CrCl_3_ when compared to the untreated samples (Fig. 2). Interestingly, the extent of DNA damage caused by CrCl_3_ (150 µM) was greater than that caused by CrO_3_ (300 µM) (Fig. 2). This was a surprise to us given that the genotoxic effects of Cr(III) was not previously appreciated as much as that of Cr(VI).

Most previous studies on chromium have primarily focused on Cr(VI) compounds because they readily penetrate the cellular membrane and can be reductively metabolized to Cr(III) [3], [21], [22]. Our previous studies on Cr(VI) and Cr(III) cytotoxicity and their effects on oxidative state of yeast cells showed that they both can enter into the yeast cells and induce cytotoxicity and oxidative stress [23]. Our comparative results on the genotoxicity of CrO_3_ and CrCl_3_ presented so far provided further evidence that CrCl_3_ can also be taken up by living cells and inflict DNA damage. Other previous reports showed that the rate of absorption of Cr(III) may be affected by several factors, including the chemical and physical properties of the compound and the primary exposure route [24]–[26]. Therefore, CrCl_3_ can function as a genotoxic compound in at least some type of cells, including the yeast and mammalian Jurkat cells used in this study. These together lend further support of a previous finding that Cr(III) compounds, including CrCl_3_, can be toxic as nutrient supplements [27].

### Both Cr(VI) and Cr(III) Induce DNA Damage *in vitro*

**Figure 3:**
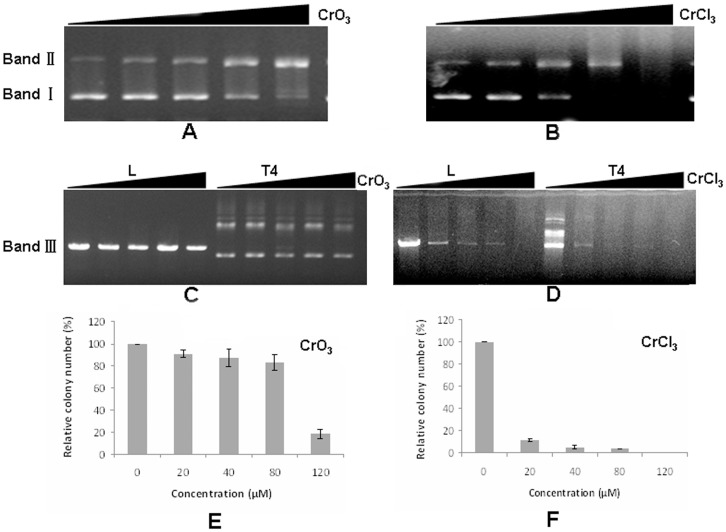
Chromium compounds induce DNA damage *in vitro*. (A, B) Plasmid DNA (25 ng/uL) was incubated with or without CrO_3_ or CrCl_3_ at increasing concentrations (0 µM, 20 µM, 40 µM, 80 µM, and 120 µM). Nicking of DNA was manifested as the disappearance of a band of faster-migrating DNA (Band I) and the appearance of a band of slower-migrating DNA (Band II) on agarose gel electropheresis. Degradation of DNA was manifested as the disappearance of both bands. (C, D) Linearized YEplac195 DNA (40 ng/uL) by Hind III and T4 ligase-treated DNA was incubated with different concentrations of CrO_3_ and CrCl_3_ and re-ligated with T4 DNA ligase. “L” indicates linear DNA and “T4” represents T4 ligase-treated DNA. Reactions were resolved with agarose gel electrophoresis. (E, F) Re-ligated DNA samples pre-treated with different concentrations of CrO_3_ or CrCl_3_ (0 µM, 20 µM, 40 µM, 80 µM, and 120 µM) were transformed into DH5α competent cells. Relative transformation efficiency was calculated and plotted.

We next investigated whether both CrCl_3_ and CrO_3_ can cause DNA damage *in vitro* using cleavage of supercoiled plasmid DNA as the assay [28]. We found that treatment with increasing concentrations of both Cr(VI) and Cr(III) leads to the disappearing of supercoiled DNA (Band I) (Fig. 3A and 3B). This was accompanied by the appearing of nicked circular or linear DNA. Eventually, all DNA molecules in a sample could be cleaved (Fig. 3A and 3B). These results suggested that both Cr(VI) and Cr(III) can induce DNA cleavage *in vitro*. To test whether Cr compounds might only interact with supercoiled DNA, we next evaluated their effects on linearized plasmid DNA. We found that linear plasmid DNA is quickly degraded upon incubation with CrCl_3_ but the effect of CrO_3_ was not as obvious (Fig. 3C and 3D). We also investigated whether the degraded DNA can be re-ligated back to circular forms to produce transforming units in bacterial cells. Such reactions were compromised when DNA molecules are pre-treated with both chromium compounds (Fig. 3C, 3D, 3E and 3F). These results together suggest that both CrCl_3_ and CrO_3_ can cleave both supercoiled DNA and linearized DNA molecules, and induce their degradation. Notably, the ability of CrCl_3_ to induce DNA cleavage and degradation *in vitro* is greater than that of CrO_3_. This observation is consistent with the *in vivo* results described in the previous section.

### Effects of Buffer and Temperature on Degradation of DNA by Cr(VI) and Cr(III)

**Figure 4:**
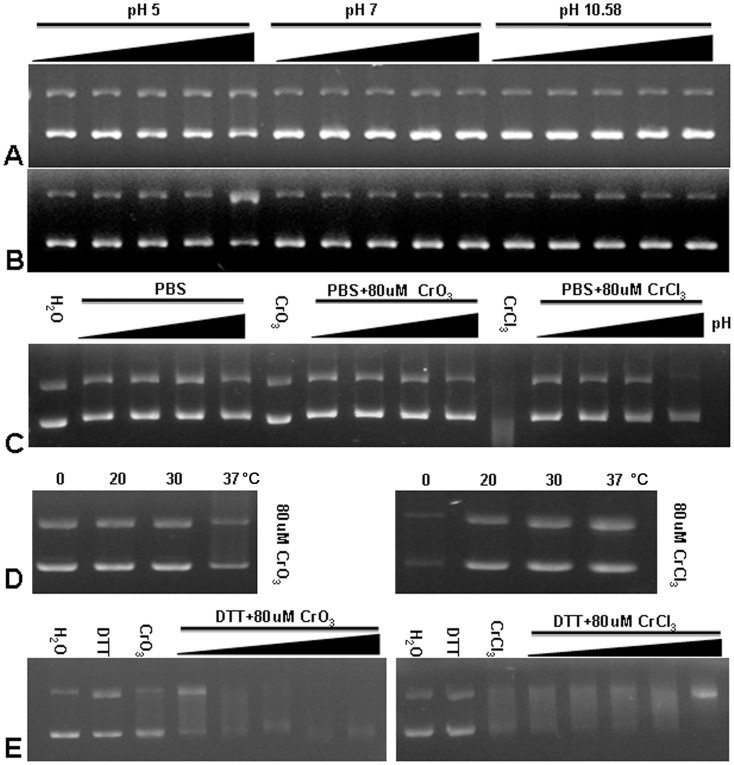
The effects of pH (A–C), temperature (D) and DTT (E) on chromium-induced plasmid DNA damadge. (A) Plasmid DNA samples were treated with increasing concentrations of CrO_3_ (0 µM, 20 µM, 40 µM, 80 µM, and 120 µM) in Tris-HCl buffers of pH 5, pH 7, and pH 10.58 and analyzed with agarose gel electrophoresis. (B) Plasmid DNA samples were treated with increasing concentrations of CrCl_3_ (0 µM, 20 µM, 40 µM, 80 µM, and 120 µM) in Tris-HCl buffers of pH 5, pH 7, and pH 10.58 and analyzed with agarose gel electrophoresis. (C) Plasmid DNA samples were treated with or without CrO_3_ (80 µM) or CrCl_3_ (80 µM) in PBS buffers of pH 4, pH 5, pH 7, and pH 10. (D) Plasmid DNA samples were treated with or without CrO_3_ (80 µM) or CrCl_3_ (80 µM) at 0°C, 20°C, 30°C, and 37°C. (E) Plasmid DNA samples were treated with or without CrO_3_ (80 µM) or CrCl_3_ (80 µM) in the presence or absence of increasing concentrations of DTT (0.1 mM, 0.5 mM, 1.5 mM, 5.0 mM and 10 mM). DTT concentration used in the absence of a chromium compound was 10 mM.

The normal pH values of the CrO_3_ and CrCl_3_ solutions are 4.18 and 4.12, respectively.We next investigated whether the effects of Cr(VI) and Cr(III) on DNA degradation might be related to the low pH of these solutions. We found that Tris buffers at pH 5, pH 7, and pH 10.58 all prevented DNA degradation by both Cr(VI) and Cr(III) (Fig. 4A and 4B, compared to Fig. 3A and 3B). From these results, it was hard to conclude whether the preventive effects are caused by Tris or by the higher pH. To investigate this further, we next tested the effects of PBS buffers at pH 4, pH 5, pH 7, and pH 10 on DNA degradation by Cr(VI) and Cr(III). Under the conditions used, PBS at all four pH values blocked or reduced DNA degradation by CrCl_3_ (at 80 uM) (Fig. 4C). However, the protective effect offered by the solution at pH 10 was smaller than that by those solutions at lower pH values. These results suggest that Cr(III)-induced DNA degradation is not caused by the low pH of the solutions.

We also investigated possible effects of different temperatures (i.e. 0, 20, 30 and 37°C) on Cr-induced DNA degradation. We found that a higher temperature (37°C) favors Cr(VI)-induced DNA degradation and a lower temperature (0°C) favors Cr(III)-induced DNA degradation (Fig. 4D). These results also suggest that the modes of action on DNA are likely different between Cr(VI) and Cr(III).

### Effects of DTT on Cr-induced DNA Degradation

Previous studies have suggested that reduction of chromate by intracellular reductants results in the formation of reactive oxidative species, which cause various DNA lesions [29], [30]. We thus next investigated whether Cr-induced DNA damage might be related to stimulation of oxidation. To this end, we tested the effects of dithiothreitol (DTT), a very strong reducing agent, on Cr(VI)- and Cr(III)-induced DNA degradation. We found that, DTT promoted DNA cleavage and degradation caused by CrO_3_ (Fig. 4E). This is consistent with the model that, when encountering reducing agents, CrO_3_ stimulates the formation of reactive oxygen species and to cause DNA damage [29], [30]. However, we also can not rule out the possibility that DTT promote DNA degradation by converting Cr(VI) to the more genotoxic Cr(III). In contrast, DTT slightly reduced CrCl_3_-induced DNA degradation at the highest concentration tested (i.e., 10 mM) (Fig. 4F). These results further suggest that Cr(VI) and Cr(III) cause DNA damage likely through different mechanisms.

### Both Cr(VI) and Cr(III) Cause Structural Changes in DNA Molecules

**Figure 5:**
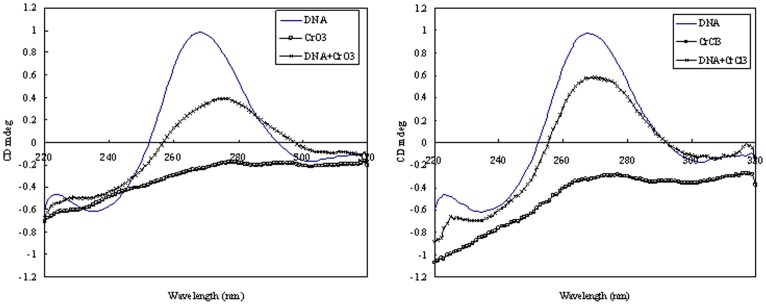
Effects of Cr(VI) and Cr(III) on the CD spectra of plasmid DNA. Plasmid DNA (25 ng/µL) was measured in the presence or absence of 150 µM CrO_3_ or CrCl_3_.

Both Cr(VI) and Cr(III) are previously shown to directly binding to DNA. We next investigated whether they might bind to DNA in different manners using circular dichroism (CD) spectroscopy. Consistent with the model that both Cr(VI) and Cr(III) directly bind to DNA, we found that they both alter the CD spectrum of DNA molecules. We also observed significant difference between the treatment of DNA with CrO_3_ and with CrCl_3_. CrO_3_ reduced the intensity of both positive and negative ellipticity bands (Fig. 5), suggesting alterations in both the stacking mode and the orientation of the base pairs in DNA molecules. This is characteristic of an intercalative interaction between a compound and DNA [31]. On the other hand, CrCl_3_ reduced the intensity of the positive ellipticity band but had little effect on the negative ellipticity band (Fig. 5), suggesting interference with the base pair stacking only. Therefore, CrO_3_ likely intercalates into the planes between the base pairs of the DNA and CrCl_3_ likely only alters the DNA stacking mode.

**Figure 6:**
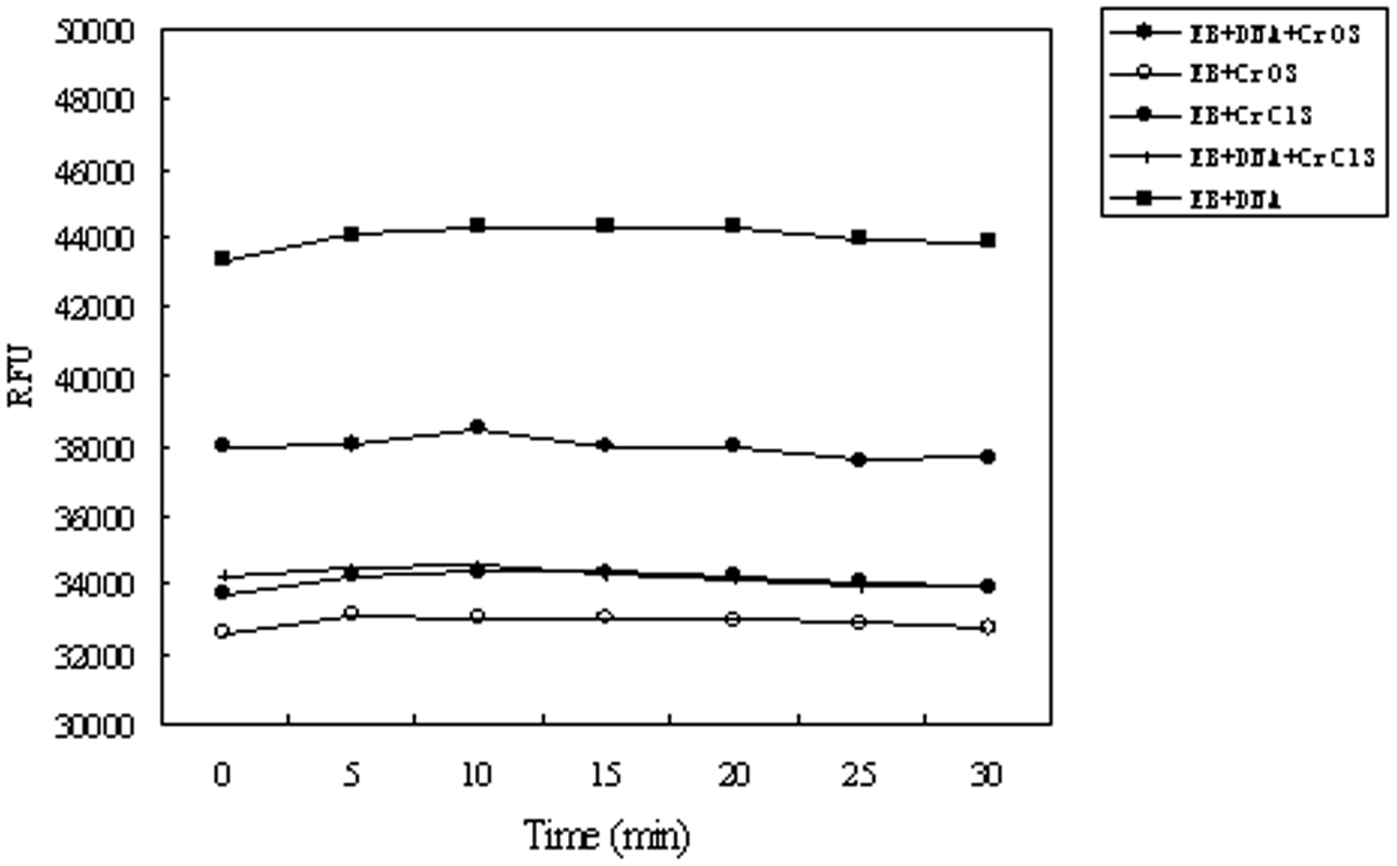
Effects of Cr(VI) and Cr(III) on binding of ethidium bromide (EB) to DNA molecules. DNA samples (6 µg/mL) were first incubated in the presence or absence of 150 µM CrO_3_ or CrCl_3_ and then with equal volume of EB (12 ug/mL). Binding of EB to DNA under each condition was measured as the relative fluorescence intensity unit (RFU).

To further corroborate these models, we measured binding of ethidium bromide (EB) to DNA molecules pre-incubated with or without Cr(VI) or Cr(III) using fluorescence spectroscopy. EB intercalates with DNA and gives rise to fluorescence emission [32]. Neither Cr(VI) nor Cr(III) caused obvious DNA degradation under the experimental conditions, yet we found that Cr(VI) only partially block EB-DNA binding, as reflected by reduced but not completely abolished fluorescence intensity (Fig. 6). This is consistent with the model that both EB and Cr(VI) intercalate DNA molecules and that they compete with each other. In contrast, Cr(III) completely blocked the binding of EB to DNA (Fig. 6), consistent with a model that Cr(III) and EB likely do not compete with each other for binding to DNA. Alternatively, these results could suggest that Cr(III) is more effective in inflicting DNA structural distortion than Cr(VI) *in vitro*.

**Figure 7:**
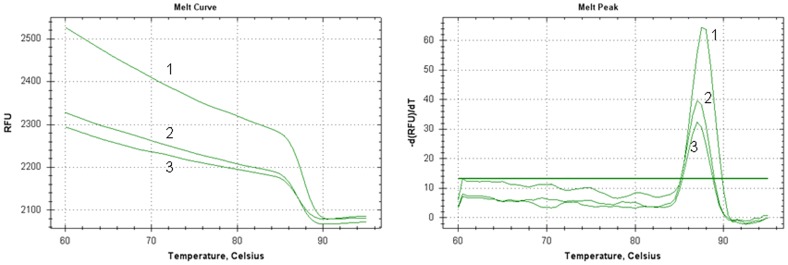
Effects of Cr(VI) and Cr(III) on *Tm*. The plotted are melting curves of a GAPDH gene fragment in the absence (curves 1) and presence of 150 µM CrO_3_ (curves 2) or CrCl_3_ (curves 3).

We also investigated possible effects of both CrO_3_ and CrCl_3_ on the melting temperature (*T*m) of DNA molecules. We did not find an obvious effect of either compound on the *Tm* of DNA (Fig. 7). However, both compounds induced hypochromicity (Fig. 7), which indicates more closed DNA structures than in the control samples. Again, the effect of Cr(III) was slightly greater than that of Cr(VI) (Fig. 7), consistent with the observations that Cr(III) is more genotoxic than Cr(VI).

In conclusion, our *in vivo* results suggest that both Cr(VI) and Cr(III) significantly induce genetic mutation in yeast and cause DNA damage within both yeast and Jurkat cells and that both can act as genotoxic compounds. In addition, the ability of CrCl_3_ to generate DNA damage is significantly greater than that of CrO_3_. The results provide unambiguous evidence for the significance of Cr(III) compounds in the generation of genotoxic damage. Our results also indicate that the mode of CrCl_3_ interaction with DNA is different from that of CrO_3_. Previous studies on chromium genotoxicity mainly focused on the formation of chromium-DNA adducts [33]–[35]. There are evidences that Cr(III) can interact electrostatically with the DNA phosphate backbone [36], [37]. Cr(III) was also suggested to form covalent bonds with phosphate on the backbone of DNA [38]–[41] and with the endocyclic nitrogen atoms of the DNA bases[42]–[46]. In this study, we demonstrated that Cr(III) likely also interferes with the stacking mode of DNA base pairs and causes DNA cleavage and degradation of DNA. Cr(VI) was previously suggested to interact with DNA via two modes of interaction: inducing structural changes and DNA compaction [11]. Our results further suggested that Cr(VI) can intercalate into the planes between DNA base pairs and cause DNA structural changes and degradation.
